# Prevalence and Lineage Diversity of Avian Haemosporidians from Three Distinct Cerrado Habitats in Brazil

**DOI:** 10.1371/journal.pone.0017654

**Published:** 2011-03-08

**Authors:** Nayara O. Belo, Renato T. Pinheiro, Elivânia S. Reis, Robert E. Ricklefs, Érika M. Braga

**Affiliations:** 1 Departamento de Parasitologia, Instituto de Ciências Biológicas, Universidade Federal de Minas Gerais, Belo Horizonte, Brazil; 2 Universidade Federal do Tocantins, Palmas, Brazil; 3 Department of Biology, University of Missouri-St. Louis, St. Louis, Missouri, United States of America; Texas A&M University, United States of America

## Abstract

Habitat alteration can disrupt host–parasite interactions and lead to the emergence of new diseases in wild populations. The cerrado habitat of Brazil is being fragmented and degraded rapidly by agriculture and urbanization. We screened 676 wild birds from three habitats (intact cerrado, disturbed cerrado and transition area Amazonian rainforest-cerrado) for the presence of haemosporidian parasites (*Plasmodium* and *Haemoproteus*) to determine whether different habitats were associated with differences in the prevalence and diversity of infectious diseases in natural populations. Twenty one mitochondrial lineages, including 11 from *Plasmodium* and 10 from *Haemoproteus* were identified. Neither prevalence nor diversity of infections by *Plasmodium* spp. or *Haemoproteus* spp. differed significantly among the three habitats. However, 15 of the parasite lineages had not been previously described and might be restricted to these habitats or to the region. Six haemosporidian lineages previously known from other regions, particularly the Caribbean Basin, comprised 50–80% of the infections in each of the samples, indicating a regional relationship between parasite distribution and abundance.

## Introduction

Understanding the influence of different landscapes on parasite-host interactions in wild populations is of particular relevance in a world undergoing dramatic change in climate. Such environmental change can influence parasite-host relationships in many ways [1], particularly by altering the transmission of endemic pathogens of wildlife, which can lead to the emergence of new diseases in wild populations, domesticated animals, and humans [2]. Habitat degradation and diversity loss due to increased conversion of natural habitats to agricultural uses and urbanization influence the distribution and abundance of wildlife species and thus may be a major driver of change in the ecology of pathogen transmission.

Increases in the occurrence of human malaria in degraded habitats have been related to habitat change altering the distribution and behavior of human malaria vectors [3], [4]. As human populations continue to grow exponentially, and as globalization increases with more travel and trade, anthropogenic pressures on wildlife habitat and populations also will increase [5].

The prevalence of malaria in natural populations of birds and mammals exhibits complex associations with landscapes over a range of spatial scales [6]–[8], including the influence of habitat degradation on vectors and disease patterns [9].

Brazil stands out among countries with high biodiversity. Thus, its natural environments are considered a priority for conservation because of their high degree of endemism and advanced degradation [10]. Nine of the ten countries that account for more than 80 percent of the world's primary forest area lose at least 1 percent of their forest area per year; Brazil loses 4 percent each year [11]. Among non-forest environments, the Brazilian Cerrado, which is considered one of the world's biodiversity hotspots [10], is also one of the most endangered ecosystems in Brazil, being rapidly reduced in area due to agricultural expansion and urbanization. Brazil has lost 48.2% of its original Cerrado vegetation. Nowadays, deforestation destroys about 20,000 square kilometers of Cerrado each year. In September 2009, the Action Plan for Prevention and Control of Deforestation in the Cerrado Lands (PPCerrado) was launched to stop this forest devastation by 2011. [12]

The impacts of human activities on the Amazon forest and Cerrado biomes are magnified in areas of transition between the two types of environment because of the importance of ecotones for the maintenance of wildlife populations [13]. The role of ecotones between major biomes for the maintenance of parasite populations is, however, poorly understood. Avian populations differ morphologically between the rainforest and ecotone, despite high gene flow; the morphological differences between habitats are as large as those found between related species [14]. In addition, because ecotone habitats may be a source of evolutionary novelty, greater attention should be paid to their conservation in order to preserve the processes that may be important to maintain rainforest diversity [15]. In the present study, we analyze the prevalence and lineage richness of haemosporidian parasites infecting Brazilian wild birds in three environments: natural cerrado habitat, urbanized cerrado habitat, and cerrado habitat that is intermixed with areas of Amazonian forest in a natural transition zone. Comparisons between these locations should provide a first indication of the influences of anthropogenic disturbance and habitat ecotones on the parasites of an assemblage of birds.

## Results

### Screning microscopy and PCR

We examined samples from 676 wild birds (29 families and 122 species) for haemosporidian parasites. Of these, 49% were infected with *Plasmodium* spp. or *Haemoproteus* spp., using both microscopy and PCR as diagnostic methods.

The proportion of infected birds detected by PCR and by microscopy differed significantly (Table 1). Considering the microscopy results as the reference point, 254 PCR-positive samples and 165 microscopy-positive results yielded a sensitivity of 72% (95% CI: 65–79%); 422 PCR-negative and 511 microscopy-negative birds yielded a specificity of 74% (95% CI: 69–76%). Some blood parasite infections amplified by PCR were not detected by microscopy (6.6%), and 19% of samples were negative by microscopy and positive by PCR. Thus, the overall agreement (95% CI) of the PCR with the microscopy standard estimated by Youden's index was 0.46 (0.38–0.54).

**Table 1:** Prevalence (%) of *Plasmodium*/*Haemoproteus* in the three different areas studied by PCR and microscopy methods.

Areas	PCR	Microscopy	PCR/Microscopy
Urban area	41	29	56.2
Lajeado State Park	29.3	20	42.2
Cantao State Park	40.1	24	48.3
**Total prevalence**	37.6	24.4	49

The combination of PCR and microscopy revealed *Plasmodium* sp. or *Haemoproteus* sp. infections in 26 families (89.7%) and 93 species (76%) of avian host. The species with the highest prevalence of infection were *Cacicus cela* (n = 8, 88%), *Formicivora grisea* (n = 7, 87.5%), *Ramphocelus carbo* (n = 12, 75%), *Manacus manacus* (n = 21, 66.7%), *Turdus leucomelas* (n = 22, 55%), *Elaenia chiriquensis* (n = 28, 50%), and *Pipra fasciicauda* (n = 112, 43%). The majority of species birds caught are resident in the region; only six migratory species (*Elaenia chiriquensis*, *Elaenia cristata*, *Myiarchus ferox*, *Myiarchus swainsoni*, *Tyrannus melancholicus*, *Vireo olivaceus*) were among those infected, *Elaenia chiriquensis* being the one with the highest prevalence.

The prevalence of haemosporidian infections varied among the three areas studied (Table 1). Prevalence in the urban area (disturbed cerrado, UA: 56.2%; n = 108) exceeded that of the intact cerrado (LSP: 42.2%; n = 76; p = 0.007). Prevalence in the intact cerrado and transition area did not differ significantly (CSP: 58.3%) (n = 147; p = 0.2). Parasite prevalence in the urban and transition areas did not differ significantly using both microscopy and PCR as diagnostic methods (p = 0.70). The analysis of a single family of wild birds (Pipridae), which had similar representation in our sample among the three areas, revealed similar prevalences (p = 0.5); haemosporidian prevalence also was not heterogeneous among areas in the sample of the single most abundant species in our sample, *Pipra fasciicauda* (Pipridae) (p = 0.5). Parasite prevalence in three additional common species (*Turdus leucomelas*, *Coereba flaveola* and *Volatinia jacarina*) did not differ between intact cerrado and the urbanized area (p>0.05).

### Parasite lineages and habitats

**Figure 1:**
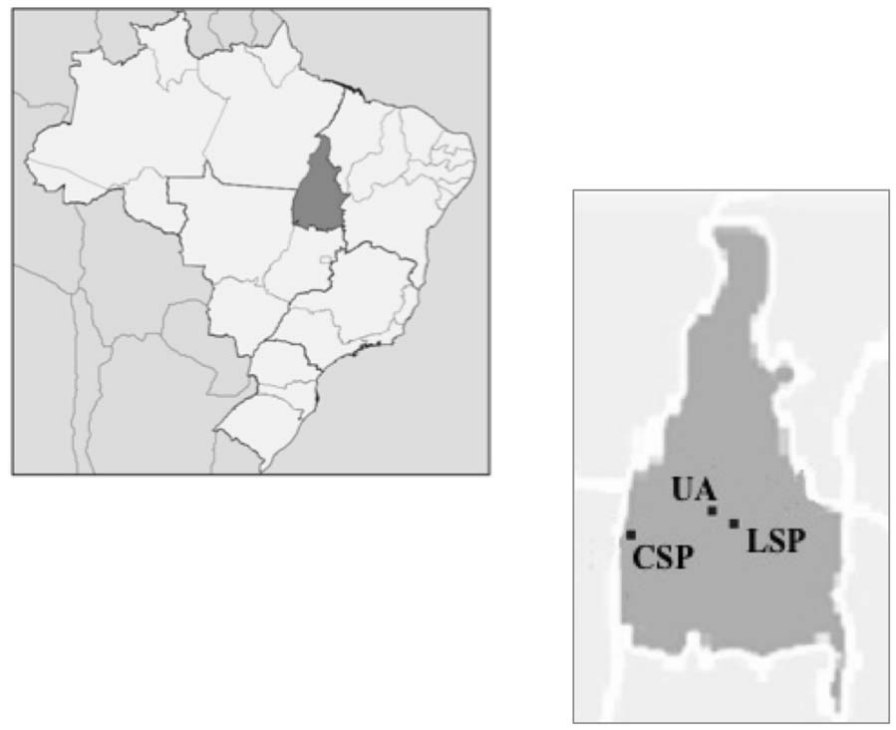
Phylogram of *Plasmodium* spp. and *Haemoproteus* spp. lineages in community birds. Phylogenetic relationships of the 21 haemosporidian parasite lineages found in three different habitats, based on cyt b sequences. Numbers located on the top of the branches indicate ML bootstrap support (100 replications, only values above 50% are shown). The presence of each parasite lineage in the three areas studied is indicated by: transition area (•), urban area (▴) and natural area (▪).

**Figure 2:**
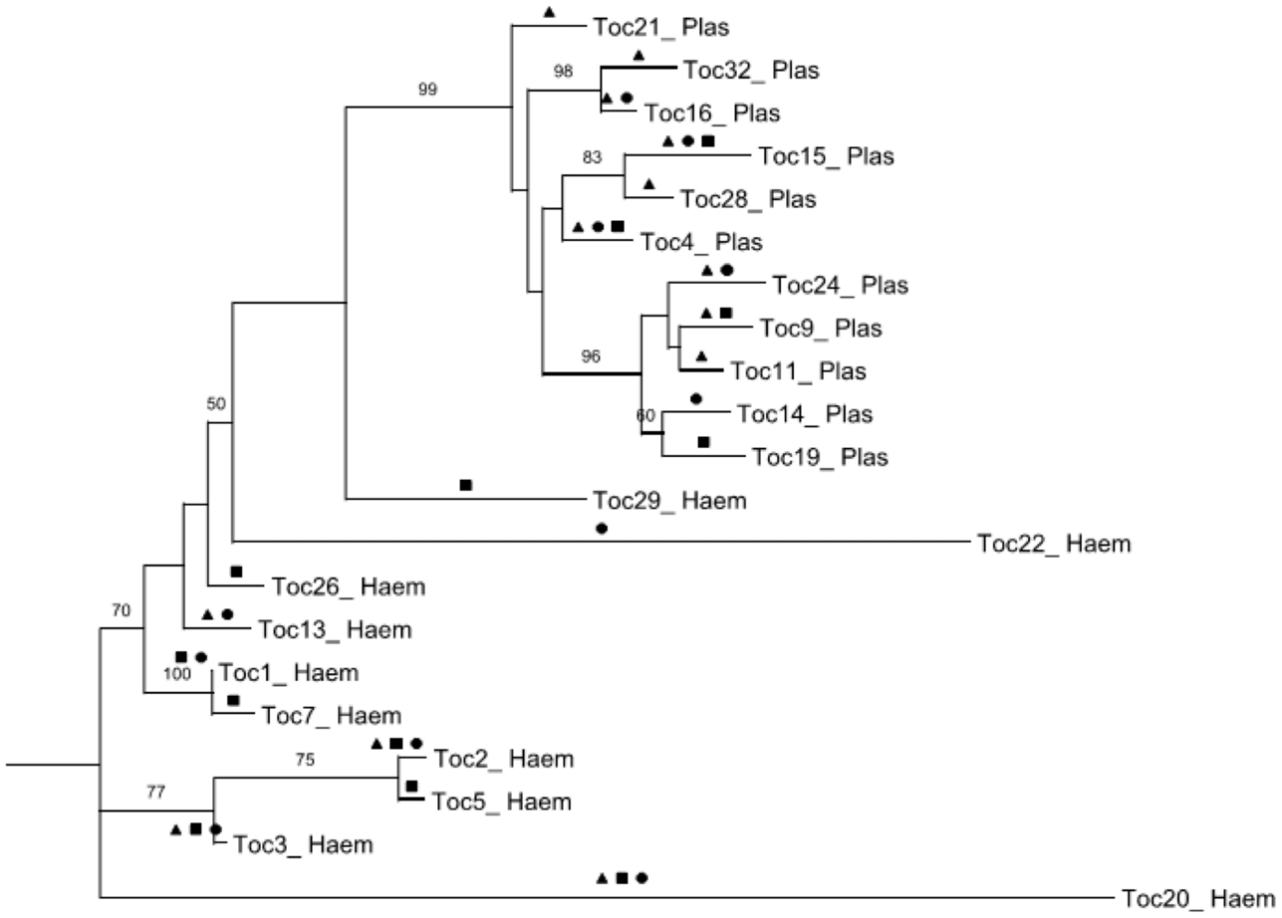
The Venn diagram above shows the distribution of the parasite lineages among the three areas, with the total number of lineages indicated for each of the three areas in parentheses, and the number of widespread lineages indicated within each of the sections of the diagram in parentheses. It is interesting that the mixed habitat area (CSP) has the fewest lineages uniquely found there.

Sequencing of the cytochrome b gene revealed 11 *Plasmodium* lineages and 10 *Haemoproteus* lineages overall. We were unable to determine sequences of 19 individuals that showed multiple infections and of some additional samples with degraded DNA. Of 11 *Plasmodium* lineages, 6 were restricted to a single study area: TOC-11, TOC-21, TOC-28 and TOC-32 in the urban area; TOC-19 in LSP; and TOC-14 in CSP (Figure 1). Five of 10 lineages of *Haemoproteus* were recovered from single areas: TOC-5, TOC-7, TOC-26 and TOC-29 in LSP; and TOC-22 in CSP. The lineages of *Plasmodium* TOC-4, TOC-9, TOC-16, TOC-24 and TOC-15 and lineages of *Haemoproteus* TOC-1, TOC-2, TOC-3, TOC13 and TOC-20 were observed in more than one habitat (LSP, CSP or/and UA) (Figure 1). Six of the lineages found in this study had previously been described in other studies (Table 2). Fifteen lineages are described for the first time in this study: 8 *Plasmodium* and 7 *Haemoproteus*. The widespread lineages were recovered from significantly more host species (6.5±5.2 sd, range 2–14, n = 6) than the local lineages (1.7±1.1 sd, range 1–5, n = 15) (Kruskal-Wallis χ^2^ = 6.8, df = 1, p = 0.01). The Venn diagram in Figure 2 summarizing the distribution of the parasite lineages among the three areas shows that the mixed habitat area (CSP) has the fewest unique lineages.

**Table 2:** Relation of parasites lineages already related in others studies.

Parasite Lineage	GenBank number Identical Lineages	Source
Toc2_ Haem	AY540204	Ricklefs RE, et al. (2004)
Toc4_ Plas	DQ659549	Beadell JS, et al. (2006)
Toc5_ Haem	AY167242	Fallon SM, et al. (2003)
Toc15_ Plas	AF465559	Ricklefs RE, Fallon SM (2002)
Toc20_ Haem	HM222483	Ricklefs RE, Outlaw DC (2010)
Toc32_ Plas	GQ395654	Levin II, et al. (2009)

Parasite diversity was higher in the urban area (78.7% of the combined diversity) than in the intact (61.4%) and transition (56.6%) areas, but the differences were not statistically significant (p>0.05) (Table 3).

**Table 3:** Parasites lineages richness of estimated by Chao1 (S) in the urban area (UA), natural cerrado (LSP) and transition areas (CSP) of the Tocantins region.

Areas	S	SD	IC 95%		%
Total Richness	22.88	2.26	21.29	32.99	100
Lajeado State Park	12.96	1.4	12.09	19.97	56.6
Cantão State Park	14.00	4.18	11.39	34.00	61.4
Urban area	18.00	5.54	13.89	41.98	78.7

SD - Standard Deviation, CI: Confidence interval. S% - Percentage of total richness estimated for each area.

**Figure 3:**
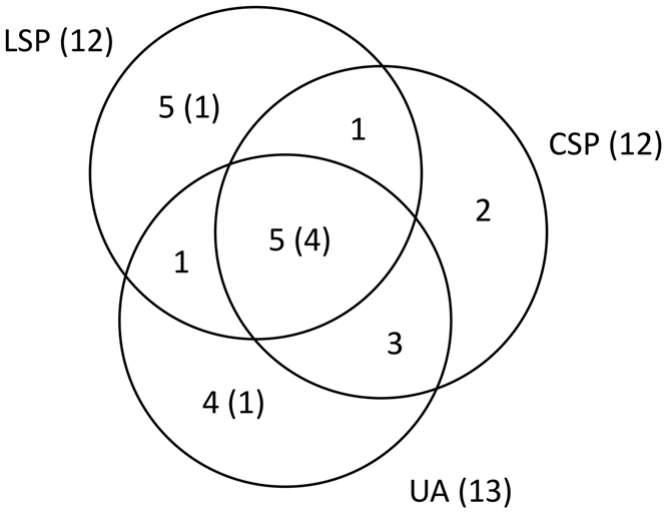
Number of host species as a function of the number of each type of parasite lineage recovered from three areas studied in Tocantins-Brazil. *Plasmodium* lineages are indicated by no fill symbols and *Haemoproteus* lineages are indicated by fill symbols. We can observer that one *Haemoproteus* lineage was presented in 21specimens from 17 different bird species. Genus is not a significant effect in an analysis of covariance (F1,18 = 2.2, P = 0.15). The equation for the line is number of hosts = −0.007 (±0.27 se) +0.77 (±0.04 se) number of infections (F1,19 = 377, P<0.0001, R^2^ = 0.96).

Table 4 lists the parasite lineages and host species and families parasitized. The number of host species increased with the number of each parasite lineage recovered (Figure 3). The proportion of parasite lineages sharing identical sequences in more than one host species occurs more in *Haemoproteus* than in *Plasmodium* (Figure 3), however these proportions did not differ significantly (p = 0.68).

**Table 4:** Host species and parasite lineages in Tocantins areas/Brazil.

Host species	Parasite Lineages (TOC)	Haemosporidian
**Galbulidae**		
*Galbula flavogaster*	2	*Haemoproteus* sp.
*Galbula ruficauda*	20	*Haemoproteus sp.*
**Bucconidae**		
*Monasa nigrifrons*	15, 22	*Plasmodium* sp.; *Haemoproteus* sp.
**Picidae**		
*Picumnus albosquamatus*	2	*Haemoproteus* sp.
**Furnariidae**		
*Dendrocolaptes certhia*	2	*Haemoproteus* sp.
**Thamnophilidae**		
*Sakesphorus luctuosus*	1, 2	*Haemoproteus* sp.
*Myrmotherula multostriata*	2	*Haemoproteus* sp.
*Formicivora grisea*	2, 24	*Plasmodium* sp.; *Haemoproteus* sp.
*Dysithamnus mentalis*	2	*Haemoproteus* sp.
*Thamnophilus torquatus*	4	*Plasmodium* sp.
**Tyrannidae**		
*Hemitriccus margaritaceiventer*	1	*Haemoproteus* sp.
*Corythopis torquatus*	3	*Haemoproteus* sp.
*Elaenia chiriquensis*	5, 15	*Haemoproteus* sp.; *Plasmodium* sp.
*Elaenia cristata*	3, 4	*Haemoproteus* sp.; *Plasmodium* sp.
*Elaenia flavogaster*	2	*Haemoproteus* sp.
*Euscarthmus rufomarginatus*	29	*Haemoproteus* sp.
*Hemitriccus striaticollis*	15	*Plasmodium* sp.
*Myiopagis gaimardii*	2,4	*Haemoproteus* sp.; *Plasmodium* sp
*Myiopagis viridicata*	14	*Plasmodium* sp.
*Myiophobus fasciatus*	26	*Haemoproteus* sp.
*Poecilotriccus fumifrons*	15	*Plasmodium* sp.
*Tyrannus melancholicus*	20	*Haemoproteus* sp.
**Parulidae**		
*Basileuterus culicivorus*	4	*Plasmodium* sp.
*Basileuterus hypoleucus*	2	*Haemoproteus* sp.
**Pipridae**		
*Manacus manacus*	2, 3	*Haemoproteus* sp.
*Pipra fasciicauda*	1, 2, 3, 4, 13, 15,16, 20, 22	*Haemoproteus* sp; *Plasmodium* sp.
**Vireonidae**		
*Vireo olivaceus*	15	*Plasmodium* sp.
*Hylophilus pectoralis*	21	*Plasmodium* sp.
**Hirundinidae**		
*Progne chalybea*	15	*Plasmodium* sp.
**Trogloditidae**		
*Cantorchilus leucotis*	3, 11, 16	*Haemoproteus* sp.; *Plasmodium* sp.
**Turdidae**		
*Turdus leucomelas*	4, 7, 9	*Haemoproteus* sp.; *Plasmodium* sp.
**Coerebidae**		
*Coereba flaveola*	1, 2, 28	*Haemoproteus* sp; *Plasmodium* sp.
**Thraupidae**		
*Dacnis cayana*	5	*Haemoproteus* sp.
*Hemithraupis guira*	15	*Plasmodium* sp.
*Neothraupis fasciata*	4	*Plasmodium* sp.
*Saltator atricollis*	19	*Plasmodium* sp.
*Saltator maximus*	9	*Plasmodium* sp.
*Tachyphonus cristatus*	2, 15	*Haemoproteus* sp.; *Plasmodium* sp.
*Tachyphonus luctuosus*	13	*Haemoproteus* sp.
*Tangara cayana*	2	*Haemoproteus* sp.
**Emberizidae**		
*Arremon taciturnus*	2, 4	*Plasmodium* sp.
*Coryphospingus pileatus*	1, 2, 4, 32	*Haemoproteus* sp.
*Volatinia jacarina*	3, 32	*Haemoproteus* sp.; *Plasmodium* sp.
**Icteridae**		
*Cacicus cela*	4, 15	*Plasmodium* sp.
*Gnorimopsar chopi*	29	*Haemoproteus* sp.

## Discussion

Several studies have addressed the geographical distribution of genetically distinct blood parasites in different regions and habitats [6]–[8], [16]–[20]. However, Neotropical areas, such as Cerrado and transition areas linking the Amazonian rainforest and the cerrado biome, have received little attention.

In the present study, we confirm the weak correspondence between PCR and microscopy results, probably due to the low-intensity of peripheral parasitemia and variation in diagnostic sensitivity [21]–[23]. In this study, the low levels of parasitemia detected by microscopic examination (ranging from 1 to 5 parasites/200 microscopic fields), with few plasmodia life stages (mainly young trophozoites), also makes morphological identification of the parasite difficult. Certainly, to obtain realistic estimates of parasite prevalence, it is important to combine both microscopy and PCR.

In our study, overall prevalence (*Haemoproteus/Plasmodium*) was higher in the urban area (urbanized cerrado, UA) and the highest number of unique *Plasmodium* lineages also was observed among birds sampled in the UA (Figure 1). We observed significantly lower prevalence of haemosporidians in the natural cerrado (LSP) compared to the UA. In contrast, we did not observe a significant difference between the diversity of parasites lineages in the natural cerrado and urbanized cerrado. However, when we analyze a single family (Pipridae) and a single bird species (*Pipra fasciicauda*) sampled in all three habitats, we observed no statistically significant difference in prevalence or parasite lineage diversity between the habitats. The presence or absence of certain host species is likely the most important factor influencing the presence of parasite lineages and overall parasite prevalence, as shown in several previous studies [24], [25]. The similarity in parasite diversity among the three areas might be related to the wide variety of wild birds distributed among these areas. Thus, it is likely that the presence or absence of a parasite lineage depends on the presence of suitable host species rather than particular attributes of the habitat independently of the host. In contrast, Chasar [8] showed that opening forest habitats in Cameroon, Africa, increased both the prevalence and diversity of parasite lineages in a particular host species. However, this type of habitat alteration likely results in more dramatic changes in avian and vector communities than the urbanization of cerrado habitat.

The high prevalence of blood parasites found in the transition area (CSP) might be related to periods of flooding, allowing reproduction of vectors and consequently increasing the number of infections. Studies have demonstrated an increased risk of malaria in humans in proximity to bodies of water, which provide abundant mosquito breeding sites [26]–[28]. Future studies of mosquito ecology may reveal the vector–parasite competence relationships in transition areas (Amazonian and Cerrado) to be particularly useful in explaining observed patterns of heterogeneity in avian malaria infection in terms of vector abundance and diversity. We have little knowledge of the vectors that transmit the parasites of these Brazil Cerrado birds. There are reports of *Culex quinquefasciatus* and *Aedes albopictus* [29], [30], which are known to be vectors of haemosporidians elsewhere, in the north of Brazil, but we do not know if these are vectors of avian malaria in the studied areas.

Spatial variation in parasite prevalence has been demonstrated [31], [32], and it is likely that different parasite lineages are associated with particular vector communities. This factor could explain the differences in the geographic distribution of parasite lineages at different sites. In this study, however, we did not observe significant differences in lineage diversity among the three habitats investigated suggesting that vectors assemblages were sufficient to support similar parasite assemblages independently of the environmental setting.

*Plasmodium* and *Haemoproteus* are widely distributed blood parasites that have been reported in many families of birds. Some studies have suggested that *Haemoproteus* parasites are relatively more host specific than *Plasmodium* [33]. However, in our study, lineages of *Haemoproteus* were shared among more host species than lineages of *Plasmodium*, although this difference was not significant. Certainly, parasites of both genera are broadly competent to infect multiple host species, which underlies the phenomenon of frequent host switching over the evolutionary history of haemosporidian parasites [16]–[19].

Habitat can influence parasite load in host species, altering the frequency of contact with vectors of blood parasites [34]. However, in this study, although we observed a greater diversity of parasites in the urban area, there was no statistical difference in the diversity of lineages among the three areas studied, including the mixed environment, which might be expected to have a high abundance of vectors following periods of flooding. Some studies at local scales [35], [36] have shown that malaria parasites may differ in prevalence and lineage diversity within small areas. However, this contrasts with the widespread distribution of most parasites in this study.

We observed broad variation in the number of hosts from which we recovered individual parasite lineages, ranging from one to 15 avian host species. The lineage with the greatest number of host species (TOC-2 *Haemoproteus* sp.) is also known from Venezuela and the state of Alabama in the United States [24]. Another lineage with wide host distribution (TOC-15, 10 hosts) is also known from Missouri in the United States and from the Lesser Antilles [24], [37]. In general, the six parasite lineages identified in this study that are also known from outside the region exhibited the greatest local abundance and host diversity in this study.

We observed individual lineages of *Haemoproteus* parasitizing many host species in as many as eight families of birds. Other surveys have detected similar host diversity in some lineages of *Haemoproteus* [38], indicating that some *Haemoproteus* lineages can infect a broad variety of hosts. In contrast to these generalist lineages, we also recovered several individual lineages from single host species. Many of these were present in low abundance in our sample, and so it is difficult to assess host distribution [24]. Additional studies in northern region of Brazil will undoubtedly expand the host ranges of many of the apparent specialist haematozoan lineages.

## Materials and Methods

### Study sites

**Figure 4:**
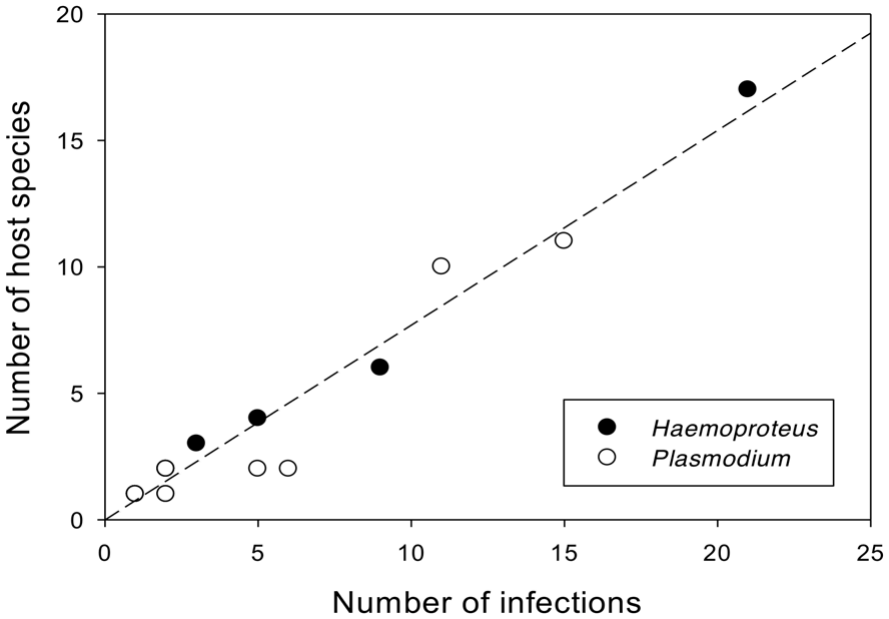
Map illustrating the collection sites in three areas studied (CSP, LSP and UA) in Brazil.

The study was carried out between 2007 and 2009 in three different areas in the Brazilian North region (5°16′N, 33°44′S) (Figure 4): 1) an urban area (UA) in the city of Palmas, Tocantins State, Brazil, consisting of cerrado vegetation being impacted by one of the highest rates of urban growth of the country; 2) Lajeado State Park (LSP), created in 2001 and located 25 km outside of the city of Palmas, preserves 9,931 hectares of natural Cerrado vegetation; 3) Cantao State Park (CSP), located in a protected area of Brazilian Amazonian rainforest and Cerrado vegetation close to the Araguaia River and including a mixture of ecosystems. The birds were caught using a constant-effort method in which the number, time, and location of capture sessions, and the time interval between sessions, were standardized. Thus, each area was sampled bimonthly with 10 mist nets operated for 12 hours during a session. The sampling was conducted during two consecutive years, covering the avian annual cycle of molt, reproduction, and post-reproduction. This method allowed us to obtain an index of the seasonal changes in populations. In the first year, the average catch per session was 71 birds; in the second, 41 birds.

We obtained blood samples from 676 birds comprising 122 species in 29 families: 190 individuals in Palmas, 181 in LSP, and 305 CSP. The bird families with the greatest number of species sampled were Tyrannidae (38 species), Thraupidae (14 species), Thamnophilidae (11 species), Dendrocolaptidae (7 species), Emberizidae (6 species) and Pipridae (4 species). The species with the greatest number of individuals sampled were *Pipra fasciicauda* (n = 112), *Elaenia chiriquensis* (n = 28), *Elaenia cristata* (n = 26), *Coereba flaveola* (n = 25) and *Volatinia jacarina* (n = 24). The study was carried out with the approval of the Universidade Federal de Minas Gerais Ethics Committee for Experimentation (Permit Number: 205/2006).

### Microscopic analysis

Blood samples were collected from the brachial vein on the underside of the wing and two or three smears were prepared using approximately 5 µL of blood. The smears were air-dried, fixed in absolute methanol, and stained for 20 min in 10% Giemsa (Sigma Chemical Co., St. Louis, Missouri, USA), pH 7.4 [39]. The slides were exhaustively examined with a light microscope, and parasite density was quantified after examination of 200 microscopic fields (approximately 150 erythrocytes/field = 30,000 erythrocytes total) at 1000× magnification under oil-immersion [39].

### Molecular analysis

#### DNA extraction

The remaining approximately 20 µL of blood sample was stored at room temperature (22–25°C) in cell lysis solution (PROMEGA, Madison, Wisconsin, USA) for approximately one day prior to DNA extraction. DNA from blood samples was extracted with the Wizard Genomic DNA Purification Kit (PROMEGA) according to the manufacturer's protocol. The DNA pellet was resuspended in 30 µL of hydration solution and kept at −20°C until use.

#### Screening

We used screening primers designed to amplify a 154 nucleotide segment of RNA-coding mitochondrial DNA [37]: 343F (5′-GCT CAC GCA TCG CTT CT-3′) and 496R (5′-GAC CGG TCA TTT TCT TTG-3′). PCR reactions were run in 10 µL volumes that contained the following final concentrations: 0.4 mM of each primer, 200 mM of each dNTP (PROMEGA), 10 mM Tris–HCl, pH 8.5, 50 mM KCl, and 1 U of Taq DNA polymerase (PHONEUTRIA, Minas Gerais, Brazil). Thermal cycling conditions were as follows: initial denaturation of 2 min at 94°C followed by 35 cycles with 1 min denaturation at 94°C, 1 min annealing at 62°C, and extension at 72°C for 1 min 10 sec. This was followed by a final extension of 3 min at 72°C. The amplified products were visualized in 6% polyacrylamide gels stained with silver nitrate [39], [40]. Microscopically negative samples showing a positive DNA amplification were exhaustively re-examined in order to confirm the presence of parasites.

#### Cytochrome b amplification

From samples in which we detected positive results (microscopy and/or mitochondrial DNA amplification), we amplified a fragment of 591 bp of the cyt b gene under the following conditions: an outer reaction using primers 3932F (5′- GGG TTA TGT ATT ACC TTG GGG TC- 3′) and DW4R (5′-TGT TTG CTT GGG AGC TGT AAT CAT AAT GTG-3′) [41] with 1 µl of genomic DNA was subjected to an initial denaturation of 4 min at 94°C, followed by 35 cycles of 94°C for 20 sec, 49°C for 10 sec, and 68°C for 45 sec, and a final extension at 68°C for 3 min. For most samples, 0.5-µl aliquot of this product was used as a template for a nested reaction with primers 413F (5′-TCA ACA ATG ACT TTA TTT GG-3′) and 926R (5′-GGG AGC TGT AAT CAT AAT GTG-3′) [25] under initial denaturation of 94°C for 1 min and 28 cycles of 94°C for 20 sec, 52°C for 10 sec, and 68°C for 50 sec and then 68°C for 7 min. PCR products were screened on 1% agarose gels, stained with ethidium bromide, and visualized with a UV light source.

#### Phylogenetic analysis

Positive PCR products were purified for cycle sequence reactions using ExoSAP-IT (USB Corporation) following manufacturer's instructions. Bi-directional sequencing with dye-terminator fluorescent labeling was performed in an ABI Prism 3100 automated sequencer (Applied Biosystems, Inc.). We sequenced 591 base pairs of the cyt b gene for *Plasmodium* spp. and *Haemoproteus* spp.

DNA sequences were aligned using CLUSTALX [42] and edited using Seq ManII version 4 (DNASTAR Inc.) and are available through GenBank (accession numbers HQ287536- HQ287556). Sequences were compared for identification to their closest matches in GenBank using the NCBI nucleotide Blast search, and to unpublished sequences using a local blast search in the laboratory of R. E. Ricklefs. We produced a maximum likelihood phylogenetic tree for the parasite sequences using RaxML [43] with the GTR + gamma model of nucleotide evolution and 100 bootstrap replications.

### Statistics

We used the nonparametric estimator Chao1 [44] calculated with scores of 200 randomizations by EstimateS 8.2 [45] to estimate the host species richness and parasite lineage richness in each study site.

Because sample sizes of hosts were unbalanced between areas, we used contingency table analyses to detect interactions between location and parasite prevalence. Statistics were carried out using Prism 5.0 for Windows (GraphPad Software, Inc.). We used Stata 9.07 to construct tables and to calculate the agreement using Youden's index (Y = Sensitivity+Specificity−1, which reflects the likelihood of a positive result among truly positive subjects versus that for negative subjects; it ranges from 0 to 1) [46] between molecular and microscopic assessments of parasite prevalence.
