# Correction: Prevalence and Lineage Diversity of Avian Haemosporidians from Three Distinct Cerrado Habitats in Brazil

**DOI:** 10.1371/annotation/993592b8-5719-45b1-814e-2052318532e7

**Published:** 2011-04-05

**Authors:** Nayara O. Belo, Renato T. Pinheiro, Elivânia S. Reis, Robert E. Ricklefs, Érika M. Braga

The legends of Figures 1-4 were out of order. Here are the correct figure legends: Figure 1. Map illustrating the collection sites in three areas studied (CSP, LSP and UA) in Brazil. Figure 2. Phylogram of Plasmodium spp. and Haemoproteus spp. lineages in community birds. Phylogenetic relationships of the 21 haemosporidian parasite lineages found in three different habitats, based on cyt b sequences. Numbers located on the top of the branches indicate ML bootstrap support (100 replications, only values above 50% are shown). The presence of each parasite lineage in the three areas studied is indicated by: transition area (●), urban area (▲) and natural area (■). Figure 3. The Venn diagram above shows the distribution of the parasite lineages among the three areas, with the total number of lineages indicated for each of the three areas in parentheses, and the number of widespread lineages indicated within each of the sections of the diagram in parentheses. It is interesting that the mixed habitat area (CSP) has the fewest lineages uniquely found there. Figure 4. Number of host species as a function of the number of each type of parasite lineage recovered from three areas studied in Tocantins-Brazil. Plasmodium lineages are indicated by no fill symbols and Haemoproteus lineages are indicated by fill symbols. We can observer that one Haemoproteus lineage was presented in 21 specimens from 17 different bird species. Genus is not a significant effect in an analysis of covariance (F1,18 = 2.2, P = 0.15). The equation for the line is number of hosts = -0.007 (± 0.27 se) + 0.77 (± 0.04 se) number of infections (F1,19 = 377, P < 0.0001, R2 24 = 0.96).

